# Polyamines: Key Players in Immunometabolism and Immune Regulation

**DOI:** 10.33696/immunology.6.206

**Published:** 2024

**Authors:** Shanmuga S. Mahalingam, Pushpa Pandiyan

**Affiliations:** 1 Department of Biological Sciences, School of Dental Medicine, Case Western Reserve University, Cleveland, Ohio, 44106, USA; 2 Department of Pathology, Case Western Reserve University, Cleveland, Ohio, 44106, USA; 3 Center for AIDS Research, Case Western Reserve University, Cleveland, Ohio, 44106, USA; 4 Case Comprehensive Cancer Center, School of Medicine, Case Western Reserve University, Cleveland, Ohio, 44106, USA

**Keywords:** Polyamines, HIV, Immune regulation, Immunometabolism, Tumorigenesis, Cancer

## Abstract

Polyamines are small organic molecules ubiquitously present in all living organisms and function as crucial regulators of biological processes ranging from fundamental cellular metabolism to immune regulation. Dysregulation of polyamine metabolism has been implicated in numerous diseases, including neurodegenerative disorders, inflammatory conditions, autoimmune diseases, and cancer. This review provides an overview of pathophysiology of these conditions, highlighting polyamines’ role in immunometabolic alterations in the context of immune regulation. Exploring the intricate mechanisms of polyamine metabolism holds promise for advancing our understanding of disease processes and developing potential innovative therapeutic interventions.

## Introduction

Polyamines are polycationic molecules containing variable hydrocarbon chains with multiple primary amino groups (-NH2) [1–4]. These occur either as diamines (putrescine, cadaverine, agmatine, 1,3-diaminopropane), polyamines (spermidine, spermine, thermospermine, caldine, thermine), or polyamine conjugates (hypusine, glutathionylspermidine) [5,6]. Polyamine synthesis essentially occurs in almost all cell types, with higher activity of the pathway in rapidly proliferating cells such as epithelial cells of the gastrointestinal tract, various cancers, neurons, glial cells (synthesized in neurons but preferentially stored in astrocytes), immune cells and reproductive cells [7–11]. Because of their cationic properties at the physiological pH, they bind to acidic sites of the cellular components including nucleic acids, membranes, and proteins [4]. They engage in a myriad of cellular functions such as modulating enzyme activities, transcription, nucleic acid synthesis/stability, RNA modifications, regulating the gene expression both at transcriptional and translational levels, protein synthesis, regulation of ion channels, kinase activities, membrane/structure functions, cellular growth, and function [4,5,12–14]. Though eukaryotic cells proficiently synthesize putrescine, spermidine, and spermine, the three major polyamines, ingestion through the diet, and production by resident microbes also serve as physiologically relevant sources [13]. Variations in their levels or obstruction in the metabolism of polyamines undoubtedly contribute to diseases including cancer, inflammation, diabetes, atherosclerosis, Parkinson’s disease (PD), Alzheimer’s disease (AD), osteoporosis, osteoarthritis, sarcopenia, renal failure, and stroke [15,16]. This review provides an overview of polyamines underscoring their roles in the context of immune cell regulation in various settings.

## Polyamine Metabolism

**Figure 1.**
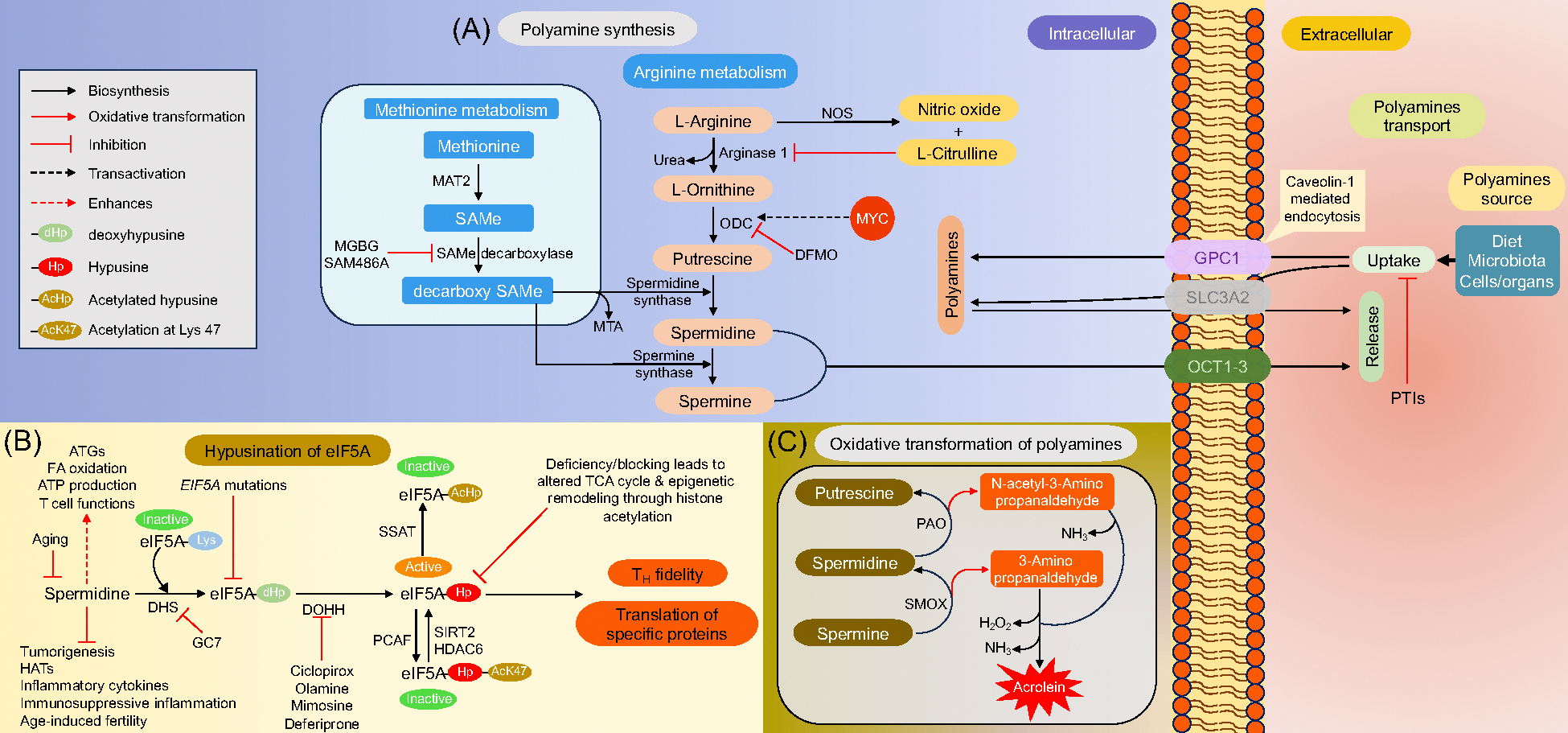
The polyamine metabolic pathways, transport, and eIF5A hypusination. Polyamine uptake by cells occurs through the cell surface proteoglycans heparin sulfate and glypican 1 (GPC1) system while putrescine transports through solute carrier SLC3A2. Spermidine and spermine efflux through the cells by ornithine transcarbamylase 1–3 (OCT1–3). The uptake could be inhibited by polyamine transport inhibitors (PTIs). **(A)** Polyamine synthesis involves two synthetic pathways either involving conversion of L-Arginine or methionine to polyamines. The first pathway involves the conversion of L-Arginine to L-Ornithine by arginase 1 with the release of urea or it is converted to nitric oxide and L-Citrulline by nitric oxide synthase (NOS). The excessive L-Citrulline acts as an allosteric inhibitor of arginase 1. Further, L-Ornithine is sequentially converted to the 3 major polyamines through a rate-limiting enzyme ornithine decarboxylase (ODC) and spermidine/spermine synthases. The ODC activity can be inhibited by difluoromethylornithine (DFMO). The proto-oncogene protein MYC functions as a trans-activator of ODC to enhance the proliferation of cancer cells and to induce tumorigenesis. The second pathway involves the conversion of methionine to polyamines through an ATP-dependent methionine adenosyl transferase 2 (MAT2) to form S-adenosylmethionine (SAMe) which is further decarboxylated to decarboxy SAMe through SAMe decarboxyalse and could be inhibited by methyl glyoxal-bis-guanidylhydrazone (MGBG) and SAM486A (CGP48664 or Sardomozide). **(B)** Multifaceted role of spermidine and hypusination of eIF5A. Hypusination occurs at a conserved lysine residue of eIF5A and is mediated by spermidine through the action of deoxyhypusine synthase (DHS) forming an inactive intermediate deoxyhypusine eIF5A which is further converted to an active hypusinated eIF5A by deoxyhypusine hydroxylase (DOHH). The active hypusinated eIF5A can be converted to an inactive form through acetylation at lysine 47 through P300/CBP-associated factor (PCAF) or by spermidine/spermine N’- acetyltransferase (SSAT) or back to its active form through deacetylation by histidine deacetylase 6 (HDAC6) or sirtuin-2 (SIRT2). **(C)** *In vitro* oxidative deamination of polyamines. The oxidative deamination causes release of toxic by-products such as aldehydes, ammonia, hydrogen peroxide and acrolein. Figure was generated using Microsoft^®^ PowerPoint.

Polyamine homeostasis is regulated by a balance between anabolism, catabolism, and the shuttling of polyamines in uni- and multi-cellular organisms [17]. The concentration of intracellular polyamines is precisely regulated *via* its *de novo* synthesis, transport, and salvage pathways that coordinate polyamine homeostasis under physiological conditions. Polyamines are largely derived from standard amino acids, arginine, and methionine in two different stages in mammals [12,16]. The first critical step involves the synthesis of ornithine and agmatine from arginine through the activities of mitochondrial arginase and arginine decarboxylase (ADC) respectively. ADC is an alternate enzyme found in nervous tissue, liver, ovary, uterus, and placenta [18]. Polyamine synthesis normally occurs through decarboxylation of its precursor ornithine by a key and a rate-limiting enzyme ornithine decarboxylase (ODC), a pyridoxal-5’-phosphate forming putrescine, which is then converted into spermidine and spermine through respective activities of spermidine synthase and spermine synthase [2,19] (Figure 1A). Hypusination is a post-translational modification that occurs in eukaryotic translation initiation factor 5A (eIF5A) protein in a conserved lysine residue, a process that requires spermidine and enzymes deoxyhypusine synthase (DHS), and deoxyhypusine hydroxylase (DOHH) [20,21]. Inactivation of DHS pathway is detrimental to eukaryotic cell growth and limits tumor progression [22–27] (Figure 1B). Synthesis of spermidine and spermine also occurs through a methionine-mediated pathway (Figure 1A) [28]. Polyamine transportation differs across the species and cell types. In bacteria, it typically involves the ATP binding cassette transporters [29]. Polyamine transport occurs *via* heparin sulfate and glypican 1 (GPC1) system or through SLC3A2, a solute carrier family of type II transmembrane protein [30–33]. In the gastrointestinal tract, putrescine uptake is mediated through nitric oxide synthase (NOS2)-dependent caveolar endocytosis in the colon and small intestine [32]. Spermidine and spermine are additionally transported by a heterospecific cation transporter known as ornithine transcarbamylase 1–3 (OCT1–3) [34] (Figure 1). The backward conversion of spermine to spermidine, and subsequently into putrescine is catalyzed by spermine oxidase (SMOX) and polyamine oxidase (PAO) respectively [1]. PAO transforms N1-acetylspermine to spermidine by the catalytic activity of spermidine/spermine-N1-acetyltransferase (SSAT) [35,36]. Under normal physiological conditions, SSAT is at negligible concentrations but could increase with elevated levels of free polyamines, regulating negative feedback [37]. Abnormal synthesis or excessive degradation of polyamines leads to extremely toxic metabolite production such as aldehydes, ammonia, peroxides and acrolein (Figure 1C). These by-products are associated with pathophysiology in mammalian systems and are more toxic than reactive oxygen species (ROS) [38–40]. This catabolic conversion involves spontaneous deamination of 3-aminopropanal through the serum PAO and spermidine oxidases to generate the toxic byproduct acrolein [41–43]. In summary, these processes underscore how excessive synthesis or degradation of polyamines could trigger abnormal cellular viability and growth.

## Methodologies Employed to Assess Polyamine Homeostasis

Current methodologies for assessing polyamine homeostasis involve HPLC (high-performance liquid chromatography), a highly reliable and sensitive method allowing for the detection of low concentrations of polyamines [44]. Techniques like LC-MS/MS (liquid chromatography-tandem mass spectrometry) provide accurate quantification and structural characterization of polyamines [45,46]. LC-MS is sensitive and specific, capable of detecting multiple polyamines simultaneously but requires specialized equipment and expertise. Also, a combination of HPLC-MS could be employed [47]. Other commonly used techniques are Western blotting and flow cytometry measuring the expression of enzymes involved in polyamine metabolism, such as ODC [48,49]. The caveats remain to be the inability to measure polyamine concentrations and variations based on antibody specificity and experimental conditions. Although RT-PCR (reverse transcription-polymerase chain reaction) is reliable for assessing gene expression, it does not provide direct measurements of polyamine levels. Metabolomics is also employed for comprehensive analysis of metabolic profiles using techniques like NMR or MS to assess polyamine levels among other metabolites which offers a holistic view of cellular metabolism but may require complex data analysis and interpretation. Each methodology has its advantages and limitations. The choice of method often depends on the specific research question and required sensitivity. Combining multiple approaches can enhance the reliability and comprehensiveness of polyamine homeostasis assessments within immune cells. Measuring polyamine levels in clinical contexts presents several challenges including sample variability arising due to diet, age, gender, health status, etc., also there are no standardized established reference values. Another important challenge would be the stability of polyamines in samples and proper sample handling and storage, which are critical for preventing degradation. Addressing these challenges is essential for the reliable measurement of polyamines in clinical practice, which could enhance their utility as biomarkers for various diseases.

## Polyamines Impact Immunometabolism

Polyamines exhibit multifaceted roles in immune cells and can influence both innate and adaptive immune responses that include modulation of (1) immune cell proliferation and differentiation, (2) macrophage polarization, (3) immune senescence and autophagy, (4) cancer immunosuppression [10,49–52]. Although polyamines were initially considered to have immunosuppressive effects, it has now become apparent that their immunobiology is nuanced and context-dependent, based on the cell types and pathological conditions. Early research highlighted the potential role of polyamines in promoting autoimmune conditions. DFMO, an irreversible inhibitor of ODC, inhibits polyamine synthesis, reduce T cell proliferation, and alleviate lupus-like disease symptoms in mice [53]. More recent work by Wagner and colleagues demonstrates that polyamines can regulate the balance between Th17 cells and regulatory T cells (T_regs_) [52]. Pathogenic Th17 cells exhibit upregulated polyamine metabolism, and pharmacological inhibition of ODC by DFMO, as well as genetic deficiency of *Odc* in mice (*Odc*^−/−^), lead to a reduction in these cells and disease severity [52]. Stimuli such as TCR activation, hypoxic conditions, stress inducers, and cytokines like IL-2, IL-17, and IL-15 promote the polyamine biosynthetic pathway in T cells. These stimuli regulate critical enzymes like ODC, SAMe decarboxylase, spermidine synthase, and spermine synthase by modulating transcription factors including c-Myc and HIF-1α. This transcriptional regulation ensures that the polyamine pathway supports T cell proliferation, differentiation, and functions differ slightly based on T cell differentiation states [47,48,54–57]. For example, polyamine synthesis can block T_reg_ fate decisions and promote autoimmunity, while spermidine alone could induce anti-inflammatory responses by promoting T_reg_ development [47]. The apparent contradictions in the results of these studies could be attributed to differences in T_reg_ subsets investigated or combined effects of polyamines evaluated in one study versus just one of the polyamines in another. They could also be due to the origin of T_regs_ from different tissues, or the concentrations of cytokines and TCR stimulants that were engaged to induce naïve cell differentiation into T_regs_, as the levels and effects of polyamines are likely different for naïve cells and differentiated T_regs_. A recent study demonstrated that deficiency in polyamine synthesis and eIF5A hypusination led to a failure of CD4^+^ T cells to adopt the correct subset specification associated with perturbations in post-translational modification of eIF5A [48]. Loss of *Odc* during the development disrupted the fidelity of Th lineage differentiation, ectopic expression of lineage-defining transcription factors, and cytokines in differentiating T cells and caused intestinal inflammation. This study shows that polyamines have another layer of mechanistic regulation of naïve T cell differentiation into various T cell subsets and may be unique to the disease-specific milieu that drives T cell polarization. They indicate a complex role for polyamines in immune regulation, with the potential for both pro-inflammatory and anti-inflammatory effects on sterile inflammation and infections.

In an infection setting, an increase in ODC and polyamine synthesis in T cells parallel with heightened ratios of PD-1^+^FOXP3^+^ IFN-γ^+^ T cells, known as T_reg_-like Th1 cells that are known to be elevated at the sites of inflammation [49, 58, 59]. HIV-infected T cells display augmented polyamine synthesis, which is dependent on ODC, caspase-1, and IL-1β activity. Blocking the caspase-1 and ODC as well as hypusination reverses T cell aberrations caused by HIV infection. Corroborating these data, salivary putrescine from people living with HIV shows a positive correlation with dysregulated T_reg_/Th17 ratio and hyperactivation of mucosal T cells [49]. T_reg_-like Th1 cells are also enriched during *Candida* infection and are physiologically relevant to infection-caused immunopathology [60]. While polyamines can favor *Candida* proliferation and altering aminopropyl group acetylation levels and autophagic induction thereby causing host cellular dysfunction, their role in T cell functions remains to be investigated in *Candida* setting [61]. These studies suggest that local polyamines in tissues have an impact on T cell subtypes and their functions in mucosal infections. Polyamines have been implicated in promoting autoimmunity, partially by regulating T_regs_ [49,60]. While polyamines can also aggravate oxidative stress and impair immune tolerance and are implicated in IBD, celiac disease, peptic ulcers, the role of T cell-polyamines in directly triggering inflammation or autoimmunity in these contexts is unclear. Mechanistically, more evidence suggest that polyamines are associated with modulating inflammation, cellular proliferation, differentiation, and mucosal healing processes in the context of immune cells. However, the direct role of T cell polyamines remains to be studied [10,62,63].

**Figure 2.**
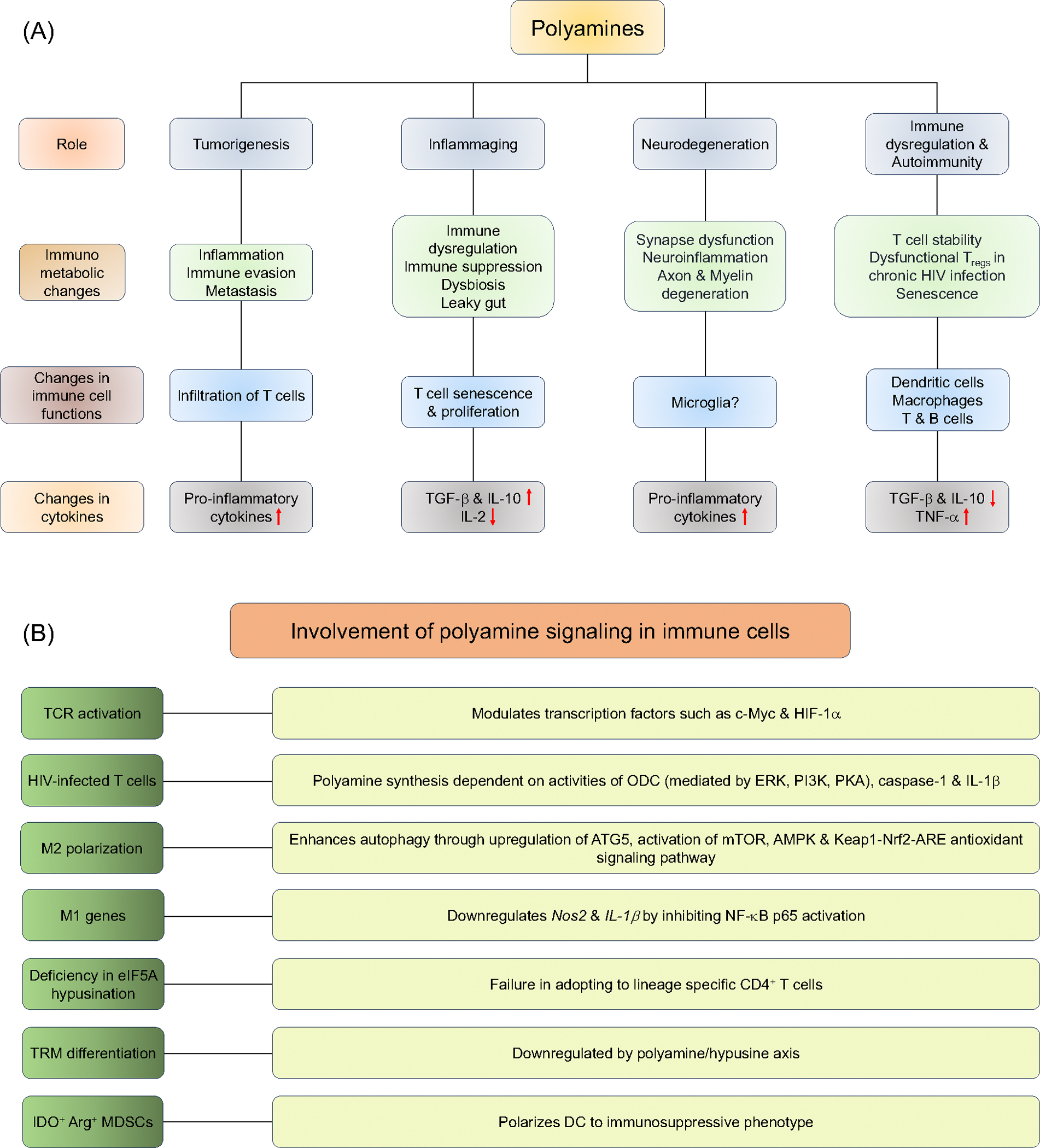
Schematic representation of pleiotropic effects of polyamine-induced immunometabolic changes and associated signaling pathways. **(A)** Polyamine immunometabolism pathways impact various cellular processes and could be manipulated by either blocking the pathway with inhibitors or uptake by polyamine transport inhibitors. Upward and Downward red arrows indicate enhanced or decreased protein expression respectively. **(B)** Modulation of polyamine-induced signaling pathways in immune cells. TCR: T cell Receptor; HIF- 1α: Hypoxia-inducible Factor-1α; ODC: Ornithine Decarboxylase; ATG5: Autophagy-related Gene 5; mTOR: Mammalian Target of Rapamycin; Nos2: Nitric Oxide Synthase 2; eIF5A: Eukaryotic Translation Initiation Factor 5A; DC: Dendritic Cells; IDO1: Indoleamine 2,3-Ddioxygenase 1; Arg: Arginase); MDSCs: Myeloid-Derived Suppressor Cells.

Polyamines are also important regulators of several other immune cells (Figure 2A). B cell receptor activation increases the levels of polyamines and the expression of enzymes involved in the polyamine pathway. The ability of spermine to regulate activation-associated apoptosis suggests a protective role for polyamines in B-cell clonal deletion processes [64]. In dendritic cells (DCs), arginase-mediated polyamine synthesis induces indoleamine 2,3-dioxygenase (IDO) expression resulting in immunosuppression. IDO signaling in DCs is dependent on arginase 1, which induces IDO phosphorylation and its activation through the activation of Src kinase. Thus, polyamines released by IDO^+^ arginase 1^+^ bystander myeloid-derived suppressor cells (MDSCs) or DCs polarize DCs towards immunosuppressive phenotype in a paracrine manner [65] (Figure 2B). Also, macrophages synthesize polyamines both through ODC and arginase pathways. The classically activated M1 macrophages which typically produce nitric oxide *via* iNOS using arginine as the substrate have reduced arginine availability for polyamine synthesis [66]. However, IL-4 and IL-13-activated M2 macrophages with upregulated arginase 1 have increased polyamine synthesis. They also show ODC-dependent polyamine synthesis mediated by ERK, PI3K, and PKA pathways independent of arginase 1 activity (Figure 2B). Polyamines appear to have diverse roles in regulating macrophage functions depending on the context. Intrinsically synthesized polyamines suppress the pro-inflammatory cytokine expression in LPS-stimulated macrophages (M1 macrophages) [67]. For example, putrescine downregulates the transcription of M1 genes (*Nos2 and Il-1β*) through the formation of euchromatin. It also inhibits the activation of M1 macrophages by inhibiting NF-κB p65 activation and downregulating TNF-α and IL-8 expression [68–70]. However, Rac1 and actin-dependent import of spermine and spermidine, which is known to increase following efferocytosis stimulate IL-1β and IL-6 expression in LPS-induced macrophages, showing pro-inflammatory effects of intracellularly imported polyamines [68]. In the context of tumor environment, spermidine either intrinsically or in a paracrine manner favors M1 polarization through *Nos2* transcription thereby inhibiting tumor growth, while spermine inhibits M1 but promotes M2 polarization, through enhancing autophagy by ATG5 upregulation [66]. Together, these studies underscore the complex and context-dependent role of polyamine metabolism in regulating T cell-mediated and T cell-independent immune responses.

Apart from the dietary arginine, gut microbiota serves as an alternative source of polyamines [13]. The gut microbiome and polyamines are intricately linked, with polyamines playing critical roles in both microbial and host physiology. Certain gut bacteria, including members of the genera *Lactobacillus*, *Bifidobacterium*, and *Enterococcus* are known to produce polyamines [71,72]. Germ-free mice exhibit significantly lower polyamine levels compared to wild type emphasizing microbiome’s role in polyamine synthesis [73]. The gut microbiota converts the dietary and host-derived amino acids to polyamines, thereby increasing local and systemic polyamine levels [74]. Resected colorectal cancer tissues from patients treated with antibiotics display decreased levels of polyamine metabolites indicating the role of gut bacteria in contributing to the human polyamine metabolite pool [75]. Therefore, disruptions in microbiome composition can alter polyamine levels, potentially affecting intestinal integrity and immune responses. Polyamines can enhance bacterial survival in the face of antibiotic treatment for an infection [76]. They bind to macromolecules targeted by antibiotics and reduce their availability for drugs [77]. They modulate outer membrane permeability in bacteria through extensive interlipid hydrogen bonding and membrane stabilization, and permeability of porin channels, impacting bacterial adhesion and colonization rates [76]. Polyamines are also shown to increase bacterial susceptibility to various antibiotics [78]. While these studies are of considerable interest, more investigations are needed to further learn how antibiotics causing dysbiosis can alter host responses by affecting microbial polyamine metabolism and how host polyamines directly affect host-microbe interactions. Gut polyamines promote the integrity of intestinal epithelium and lessen the macrophage pro-inflammatory cytokine production [79]. Several studies have demonstrated that polyamine administration improves the health of intestinal resident mucosal immune cells [13,47,80]. However, there is still much to uncover in terms of the biological mechanisms of mucosal polyamines and the full extent of their actions in the context of the microbiome. There could also be intrinsic differences in polyamine effects on mature or pathological Th subsets, and newly activated naïve cells in normal versus infection settings. These contextual changes may also be attributed to the levels of intracellular polyamines versus extracellularly available polyamines and the balance between synthesis enzymes and polyamine oxidases. Because polyamines interact with multiple cellular targets, it is challenging to narrow down their specific roles in different biological contexts. However, unraveling key mechanisms could lead to a deeper understanding of how polyamines contribute to intestinal health, immune regulation, and disease pathogenesis.

## Polyamines in Immune Regulation and Neuroinflammation

Immune dysregulation may lead to inflammation in the central nervous system and is implicated in the pathogenesis of neurodegenerative diseases such as AD, PD, and age-related cognitive decline [81,82]. Immune cells in the brain, such as microglia, play a key role in neuroinflammation by producing inflammatory mediators in response to various stimuli, including misfolded proteins and oxidative stress [83].

Spermidine can effectively act as a free-radical scavenger and curb the excessive generation of ROS including superoxide, hydroxyl, peroxyl radicals, nitric oxide, peroxynitrite, etc [84]. It also reduces the malondialdehyde formation, an indicator of lipid peroxidation [85,86]. Spermidine is also known to activate mTOR and AMPK pathways and delay brain aging through autophagy induction. Along with the enhanced expression of neurotrophic factors, it also enhances the antioxidant enzyme (activating Keap1-Nrf2-ARE antioxidant signaling pathway), maintains energy of neurons (through mitochondrial ATP production), inhibits apoptosis (by activating autophagy) and limits inflammation (by inducing M2 macrophages) [87,88] (Figure 2B). Optimal polyamine levels are crucial for neuronal replication and maintenance and are synthesized in neurons and shuttled by glial cells [89]. Agmatine produced in the brain, acts as a neurotransmitter and suppresses excessive nitric oxide production, mitigating hypoxic-ischemic brain injury in neonatal rats [90–92]. Spermidine and spermine enhance glutamate and glycine binding to NMDA receptors. The binding of spermidine and spermine acts as a positive allosteric modulator of NMDA receptors thereby enhancing glutamate and glycine binding while putrescine inhibits the process [93,94]. Since these are ionotropic receptors crucial for synaptic transmission and plasticity in the central nervous system, the exogenous addition of putrescine induces potent convulsion and neuropathological lesions in rodents. Therefore, systemic injection of a high dose putrescine (more than the physiological dose of 200 mg/kg) is known to induce a characteristic toxic response in rats [95]. Spermidine inhibits cellular senescence in neuronal cell lines through modulating mitochondrial functions and is known to improve the cognitive functions in mice and Drosophila [96–99]. However, excessive production of putrescine in the brain is known to be involved in seizures [100]. Also, AD is associated with significantly heightened spermidine and spermine levels [101–103]. Cellular polyamines are capable of binding to beta-amyloid (Aβ) plaque peptides and further promote their aggregation and successive memory loss [104]. Moreover, arginine deprivation due to hyperactivation of arginase can also contribute to neurodegeneration [28,105]. Arginine deprivation promotes oxidative and chronic maladaptive polyamine stress response (PSR), thereby accelerating the conversion of ornithine to putrescine, elevating polyamine levels and igniting the neurodegeneration cycle. Additionally, the expression levels of SSAT and PAO are high in the AD brain, suggesting a predominant role for polyamine oxidation in the neurodegenerative process. Thus, glutamate receptors, calcium dynamics, PSR, and polyamine oxidation play crucial roles in and AD pathogenesis [105–109]. Polyamine pathway alteration also leads to the aggregation of α-synuclein in intraneuronal inclusions and cellular dysfunction in PD [110]. Mechanistically, loss of function in the lysosomal polyamine exporter ATP13A2 is responsible for the accumulation of lysosomal polyamines and reducing their availability to mitochondria thereby impacting the mitochondrial functionality [110]. Cells with ATP13A2 deficiency have a higher pH and compromised degradative capacity and the addition of spermine leads to the rupture of lysosomes, release of cathepsin B, and neuronal cell death [111]. While higher acetylated polyamines are reported in the serum, putrescine levels are increased in the cerebrospinal fluid of PD patients, with those exhibiting worse phenotypes showing drastically reduced spermidine levels [112,113]. However, the functional significance of these studies is unclear. Given the significant role of T cells in neuroinflammation [114,115] (reviewed elsewhere) it is tempting to speculate how aforementioned polyamine-dependent T cell dysfunction could contribute to neuroinflammation, although the studies on exact interactions between T cells and neuronal cells in the context of polyamine metabolism remain to be done. However, modulating polyamine metabolism presents a potential therapeutic strategy for managing neuroinflammation.

## Polyamines as Immunomodulators and Molecular Drivers of Tumorigenesis

Polyamines, also function as oncometabolites, promote immune suppression, and are often correlated with tumor growth and progression [69,116]. Also, as reviewed elsewhere, tumor-derived polyamines favor M2 macrophages and other immunosuppressive cells favoring further tumor growth [117]. Paracrine effects of M2 macrophage-derived polyamines suppress the activity of T cells and DC and modulate tissue repair and remodeling by promoting the proliferation and migration of fibroblasts and endothelial cells in the tumor microenvironment [67]. Increased tumor polyamine synthesis also decreases IL-12 and IFN-γ levels in immune cells ultimately inhibiting NKT cell’s cytotoxic functions. Moreover, polyamines also contribute to tumor progression by directly enhancing cell adhesion, ECM remodeling, and angiogenesis. A direct relationship between ODC, a transcriptional target and a trans-activator of the *MYC* is well established [57,118] (Figure 1A). Therefore, polyamine-associated cancer cell growth may involve MYC signaling pathway in a wide range of cancers. Elevated ODC activity, polyamine biosynthesis, and high uptake of polyamines in tumor cells, particularly in rapidly growing tumors are not only considered prognostic markers for cancers but also pro-tumorigenic [116,119]. Studies have reported that tumor cells have elevated polyamine levels due to increased synthesis and uptake/transport with reduced catabolism. Additionally, cancer cells dependent on constant, higher intracellular levels of polyamine pools to provide persistent proliferation [1]. Several oncogenes including *MYC*, *KRAS*, *JUN*, *BRAF* and *FOS* are associated with polyamine dependent cellular dysregulation [120–122]. Furthermore, genetic polymorphisms in *ODC* are often associated with some neuroblastomas, colorectal, gastric, prostate, and breast cancers [123, 124]. Blockade of ODC activity in intestinal epithelial cells inhibits TGF-β1 mediated-SMAD signaling pathway and reduces tumor growth and vascularization [125,126]. Also, DFMO blocks the activation of p38, ERK, and AKT/mTOR/p70S6K induced by N-nitrosomethylbenzylamine in a rat model of esophageal squamous cell cancer [119]. Owing to its low toxicity and oral administration, DFMO has been used in several clinical trials to treat various cancers such as lung, pancreatic, neuroblastoma, endometrial, gastric, and osteosarcoma [127,128]. Because polyamine oxidative catabolic products and oxidative stress can enhance ODC activity and tumor cell growth, these byproducts have also been implicated in various cancers, including head and neck, gastric, lung, colorectal, breast, and prostate, as well as in several other cancer cell lines [116,119,129]. Hypusinated eIF5A is required for the malignant transformation into lymphoma in MYC-overexpressed B cells [130]. In the context of immune cells in the tumor microenvironment, polyamines enhance Ca^2+^ accumulation in mitochondria thereby promoting T cell activation [131,132]. A recent study by Al-Habsi and co-workers demonstrated that reduced levels of spermidine in CD8^+^ T cells from aged mice correlated with their non-responsiveness to PD-1 antibody therapy [133]. Spermidine supplementation, likely by its ability to bind to the mitochondrial trifunctional protein, a β–oxidation enzyme, and increase ATP production in CD8^+^ T cells, enhanced the anti-tumor immunity. Polyamine/hypusine axis, however, downmodulates tissue-resident memory T cell (Trm) differentiation [134]. Inhibition of this axis enhances IFN-γ and TNF-α production upon activation of both mouse and human CD8^+^ T cells and increases TGF-β induced differentiation of CD69^+^CD103^+^ Trm cells. Hypusination of eIF5A induces transcription of mitochondrial genes and when disrupted, greatly reduces oxygen consumption by modulating oxidative phosphorylation and mitochondrial functions in the macrophages [131]. The reasons for these disparate effects of polyamines in cancer in relevance to their direct effects on tumors versus indirect effects on immune cells and immunosurveillance versus regulation are to be explored in the future.

Several clinical trials are underway targeting polyamine pathways. The inclusion of DFMO reduced the tumor size in a mouse xenograft model of neuroblastoma [31]. A recent phase II clinical trial concluded that high-risk neuroblastoma patients receiving DFMO showed a substantial increase in overall survival compared to subjects without DFMO treatment. Earlier addition of DFMO in the maintenance therapy in amalgamation with immunotherapy and cis-retinoic acid could potentiate the therapeutic effect [135]. This has led to the initiation of a number of clinical trials NCT02395666, NCT04301843, NCT02679144, NCT02395666, NCT05717153 [135,136]. Furthermore, blockade of polyamines primes an increase in infiltration of CD8^+^ T cell and an inflammatory phenotype in tumors [137–141].

Synthetic polyamine analogs are being created to disrupt polyamine function, offering potential treatments for autoimmune conditions [1,142]. However, challenges such as systemic toxicity, the complex role of polyamines in immune regulation, and compensatory cellular mechanisms need to be addressed. More research is required to develop targeted therapies that selectively modulate polyamine metabolism in various diseases without affecting healthy cells and normal physiological functions.

## Concluding Remarks

Recent research underscores the pivotal role of polyamines in immune regulation, revealing that they enhance the activation and proliferation of T cells while influencing the function of macrophages and dendritic cells [47–49,52,56,59,117]. Overall, dysregulation of polyamine metabolism can influence disease progression through several mechanisms. Polyamines, particularly spermidine and spermine, are crucial for cellular growth and differentiation. Elevated levels can promote cell proliferation and inhibit apoptosis, contributing to cancer progression by allowing malignant cells to thrive [143,144]. Polyamines stabilize nucleic acids and modulate transcription factors, affecting the expression of genes involved in cell cycle regulation, survival, and inflammation [145,146]. The resulting immune cell changes and heightened recruitment of immune cells that suppress anti-tumor responses by supporting MDSCs, macrophages and T_regs_ could affect the tumor microenvironment by promoting angiogenesis and immune evasion [69]. Additionally, polyamines interact with gut microbiota, impacting systemic immune responses and speculating connections between gut health and autoimmune conditions [13,147]. Moreover, targeting polyamine metabolism with inhibitors such as DFMO is emerging as a promising therapeutic approach, particularly for cancer treatment and immune modulation. Recent research suggests that polyamines may also be involved in immunosenescence, affecting immune cell functionality in older adults through autophagy [96,148,149]. Collectively, these findings emphasize the importance of polyamines as critical regulators of immune responses and potential therapeutic targets in various diseases.

## Future Research Directions and Unanswered Questions

The field of polyamine immunobiology continues to evolve, with ongoing discoveries shedding light on new aspects of their involvement in regulation of immunometabolism and leading to therapeutic interventions. Several questions remain unaddressed: 1) How can therapies be designed to selectively target polyamine metabolism in immune cells in the context of above diseases and immunometabolism? 2) How do polyamines precisely regulate immunometabolism, and how do metabolic shifts in immune cells influence outcomes in different disease environments? 3) How do polyamines produced by gut microbiota interact with host immune regulation and contribute to mucosal diseases? 4) What are reliable biomarkers to monitor polyamine activity and guide therapeutic interventions? 5) How do cells develop resistance to therapies targeting polyamine metabolism, and what compensatory pathways are activated? 6) What is the optimal timing and dosage for polyamine-targeted therapies in various diseases? Addressing these queries will further advance our understanding of polyamine-mediated immune processes and unlock new therapeutic strategies for treating cancers, autoimmune, and inflammatory diseases.
